# Modified Puestow Procedure for Chronic Pancreatitis in an Adolescent Female

**DOI:** 10.7759/cureus.25503

**Published:** 2022-05-30

**Authors:** Ravi Maharaj, Aaron Haralsingh, Juliana Mohammed, Keshan Ramnarace, Hilary Lee-Cazabon

**Affiliations:** 1 Department of Clinical Surgical Sciences, Faculty of Medical Sciences, University of the West Indies, St. Augustine, TTO; 2 Department of Surgery, Eric Williams Medical Sciences Complex, St. Joseph, TTO

**Keywords:** pediatrics, general surgery, small bowel obstruction, chronic pancreatitis, puestow procedure

## Abstract

Chronic pancreatitis is an inflammatory condition resulting in fibrosis and consequent destruction of pancreatic tissue and loss of exocrine and endocrine function. Despite being an uncommon disease in adults, its incidence in children is significantly lower. Crucial surgical intervention is considered in pediatric cases where pain management and reducing the risk of future cancer development are of concern. The efficacy of the Rochelle-Partington modification of the Puestow procedure in remedying chronic pancreatitis has shown satisfactory long-term results, especially in pediatric cases, however, not without side effects.

A 13-year-old girl who suffers from recurrent abdominal pain attributed to chronic pancreatitis underwent the Rochelle-Partington modification of the Puestow procedure to mitigate her symptoms. The postoperative course was complicated by small bowel obstruction necessitating revision of the enteroenteric anastomosis. After three years since surgery, the patient remains pain-free, well-nourished, and leads a normal life without the interruption of her daily activities.

While still left to be seen if the modified Puestow procedure serves to be the superior choice in the treatment of chronic pancreatitis, it remains a safe choice for surgical treatment among adolescents. Sustaining pancreatic function is essential in pediatric cases where the long-term quality of life is concerned to reduce chronic pain and maintain nutrition.

## Introduction

Chronic pancreatitis is an irreversible process resulting in fibrosis of the pancreas due to a plethora of etiologies that can precipitate exocrine and endocrine insufficiency. This can manifest as abdominal pain, diarrhea, diabetes mellitus, and malnutrition [1]. In childhood, it is a rare condition, with an incidence of about 0.5 per 100,000 persons per year. It is a poorly understood disease entity in children, often presenting with intractable abdominal pain requiring frequent hospitalization [2]. Genetic mutations and anatomic anomalies seem to predispose to the development of both acute recurrent pancreatitis and chronic pancreatitis in both children and young adults [3].

Magnetic resonance cholangiopancreatography (MRCP) is a valuable tool reserved for investigating anatomical abnormalities which may lead to pancreatitis in the pediatric population. As conservative and medical therapies frequently fail, surgical intervention is often necessary [4,5]. The use of the Rochelle-Partington modification of the Puestow procedure in the management of chronic pancreatitis in children has been reported with good long-term success but not without complications.

## Case presentation

A 13-year-old girl suffered from recurrent upper abdominal pain for seven years due to chronic pancreatitis. The severity of her symptoms resulted in five prior hospital admissions and many lost school days. On her first admission, at the age of six, she was diagnosed with acute pancreatitis according to the Atlanta criteria, having demonstrated biochemical and radiological evidence of this disease. She was otherwise well, with no comorbidities and no prior surgeries. There was no family history of pancreatic disease or any history of alcohol or drug use.

**Figure 1:**
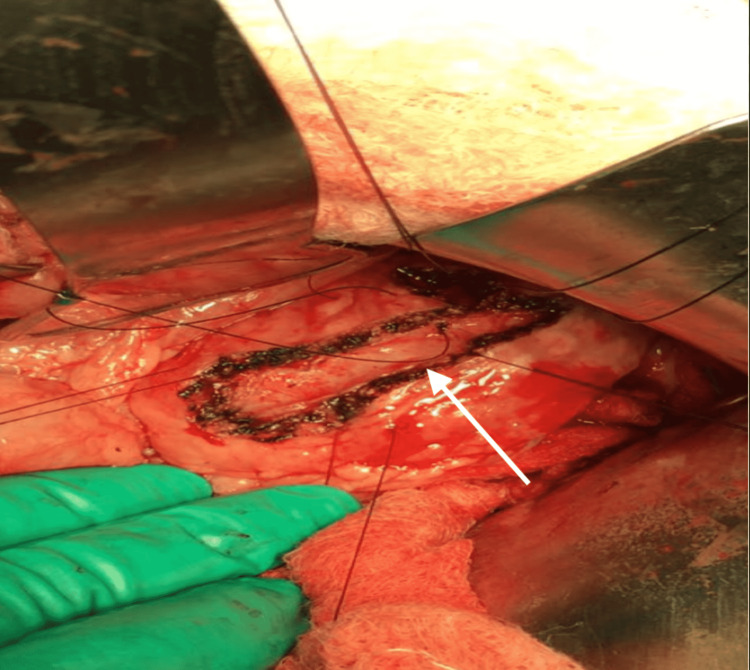
The pancreatic duct (white arrow) is opened longitudinally in preparation for jejunal anastomosis

**Figure 2:**
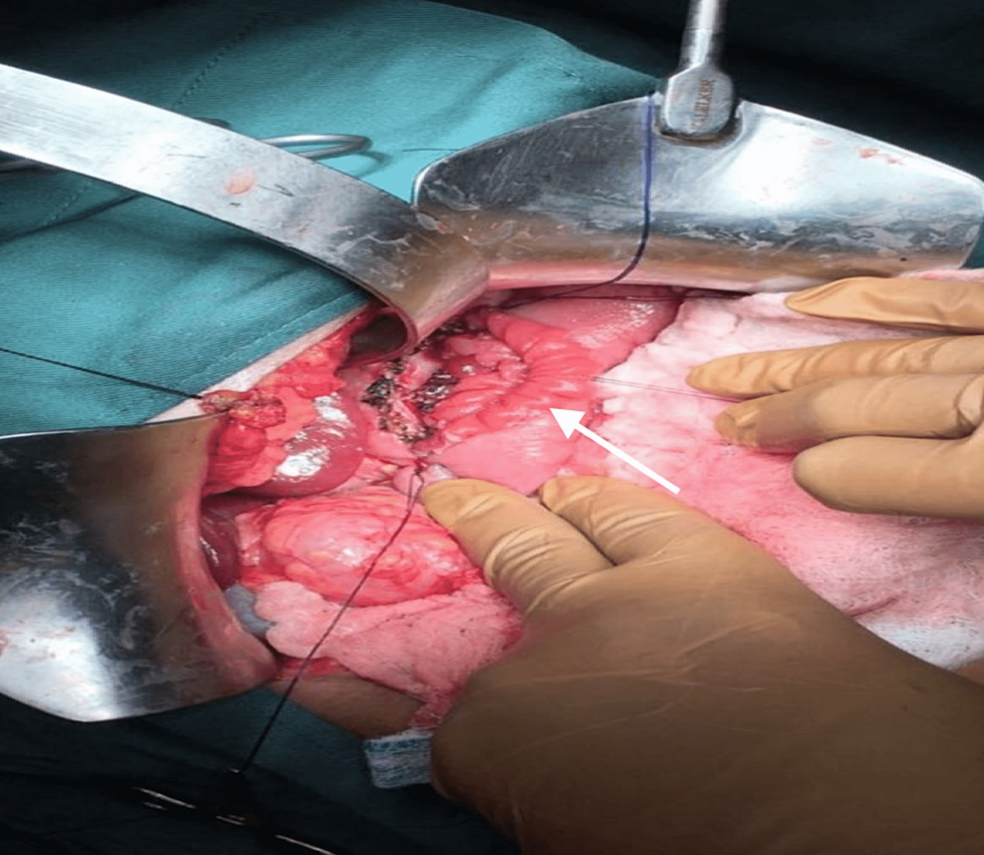
Posterior wall of Roux-en-Y lateral pancreatojejunostomy complete with 3/0 PDS—White arrow illustrates the Roux limb of the jejunum PDS: polydioxanone sutures

**Figure 3:**
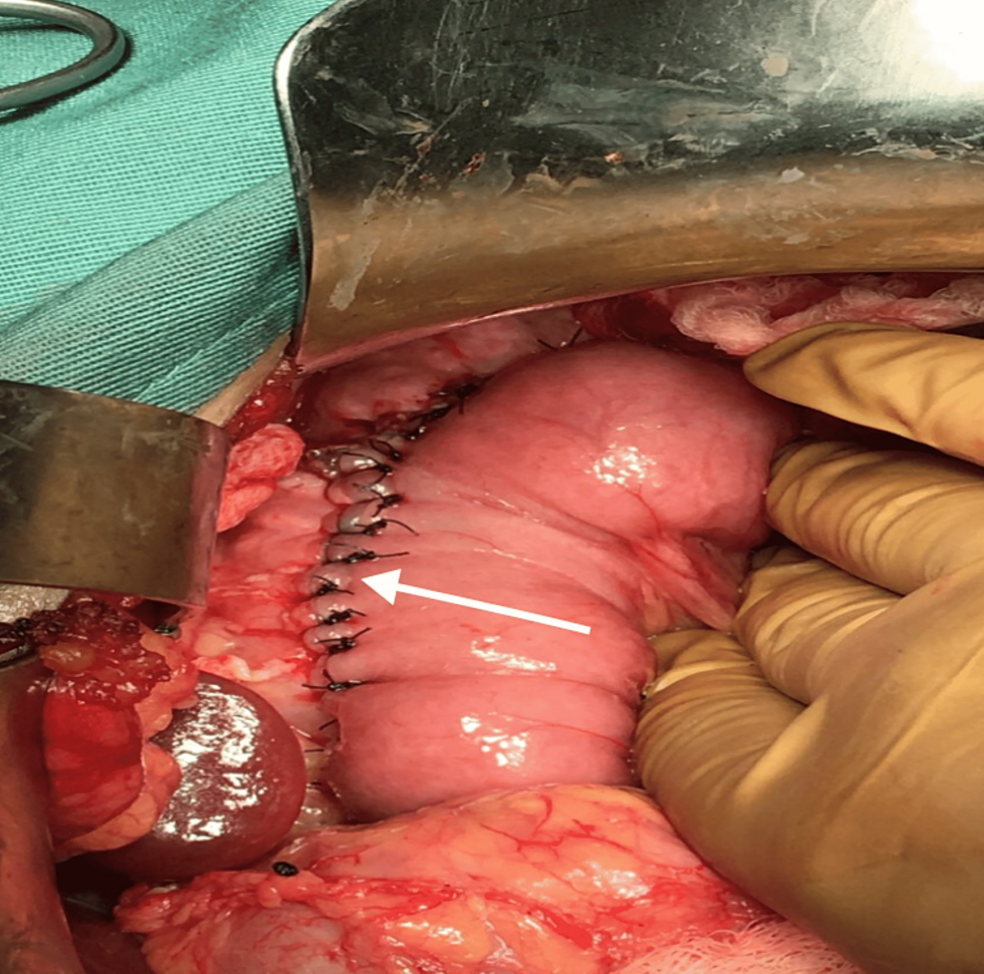
White arrow shows completed anastomosis with PDS PDS: polydioxanone sutures

Medical causes of pancreatitis, including autoimmune and metabolic disorders, were excluded after extensive investigations by the pediatric gastroenterology team. Genetic testing was not readily available at our institution. However, MRCP revealed multiple intraductal calculi with pancreatic duct dilatation throughout its course. There was no evidence of any congenital anomalies in the pancreas. The maximal duct diameter was 10 mm, near the head of the pancreas, while the maximal stone diameter was 4 mm. There were neither inflammatory changes nor was there any evidence of a pancreatic mass. The patient was diagnosed with grade 3 chronic pancreatitis as per the Cambridge Classification. Pediatric endoscopic ultrasound and endoscopic retrograde pancreatography are unavailable in our institution. To alleviate pain in the long term, the decision was made for surgical intervention. The Rochelle-Partington modification of the Puestow procedure was performed via a Chevron approach, the pancreatic body was exposed via the gastrocolic omentum. The main pancreatic duct was identified with the use of intraoperative ultrasound and the duct opened longitudinally (Figure 1). The jejunum was transected 20 cm from the duodenojejunal flexure and a 40 cm Roux loop was brought up in a retrocolic fashion to facilitate a side-to-side pancreaticojejunostomy (Figures 2-3). A drain was placed in the splenorenal fossa.

After surgery, she was nursed in the intensive care unit and recovered well until she developed bilious vomiting and peri-umbilical pain on the sixth postoperative day. Abdominal radiography did not reveal any dilated small bowel suggestive of obstruction, however, elevated drain amylase and lipase levels more than three times the serum level with an output over 100 ml daily and the need to discontinue oral intake suggested the development of a grade B postoperative pancreatic fistula. Conservative management was employed with total parenteral nutrition and octreotide forming the core of the treatment strategy. Two weeks later, she developed absolute constipation with worsening abdominal pain and contrast studies revealed that there was no flow in the common limb.

An exploratory laparotomy was performed, and it was noted that the supracolic compartment was “frozen”, while extensive adhesions were found distal to the jejuno-jejunal anastomosis. A loop of jejunum distal to the obstruction was used to perform a side-to-side anastomosis with the Roux limb. However, fistula drainage continued despite this measure, and three weeks later abdominal ultrasonography confirmed small bowel obstruction. On re-exploration, further adhesiolyis was performed, however, an on-table contrast study confirmed that there was still no distal flow. A third anastomosis was therefore created between a distal segment of the common limb and the Y-limb. Once the obstruction was relieved, resolution of the pancreatic fistula soon followed. Three years after her initial surgery, she remains well with minimal pain and no further disruption of her normal daily activities.

## Discussion

Chronic pancreatitis leads to progressive pancreatic parenchymal tissue loss which results in fibrosis, inflammation, and exocrine and endocrine loss of function [6]. In childhood, it is a rare and debilitating disorder, often refractory to medical therapies [2]. Chronic or recurrent abdominal pain is the most common clinical feature, resulting in frequent emergency room visits and hospitalizations, endoscopic or surgical procedures, and lost school days [3]. An increase in the pancreatic ductal pressure secondary to obstruction, coupled with inflammation of the peripancreatic plexus leads to refractory abdominal pain in these patients [1].

It is frequently associated with genetic mutations in PRSS1, SPINK1, and CFTR and obstructive risk factors such as gallstones and anatomical pancreatic anomalies [3]. Our patient suffered abdominal pain intermittently for seven years, had five prior hospital admissions, and failed medical therapies. The major causes of chronic pancreatitis in children, including gallstones, congenital anomalies, infection, medications, and trauma were excluded. Genetic testing is unavailable in the public healthcare system of Trinidad and Tobago at this time, so it cannot be ruled out in this case.

The International Study Group of Pediatric Pancreatitis: In Search for a Cure (INSPPIRE) consortium defines chronic pancreatitis as the presence of typical abdominal pain, exocrine insufficiency, or endocrine insufficiency plus imaging findings [4]. Our patient met the first of these criteria. Transabdominal ultrasound is often the first-line imaging for children with suspected pancreatitis. However, MRCP is the recommended imaging modality for the evaluation of the biliary and pancreatic ducts [5,6]. In our patient, imaging revealed pancreatic ductal dilatation with multiple calculi. Pancreatic ductal dilatation is the most common finding on MRCP in pediatric chronic pancreatitis. Intraductal calculi, however, are uncommon [7].

Treatment is dependent on the cause and should provide effective pain relief, improve or maintain quality of life, and preserve endocrine and exocrine function. Decompression of the pancreatic duct proximally can be done by either endoscopic retrograde cholangiopancreatography (ERCP) intervention or surgical decompression with pancreaticojejunal anastomosis [8]. Medical management revolves around pain control with non-opioid analgesia and addressing malnutrition with pancreatic enzyme replacement therapy [5]. ERCP is a viable option for pancreatic duct dilatation secondary to strictures or calculi. The success of endoscopic treatment is a predictor of good surgical outcomes later in the course of this disease. Factors influencing the decision for surgical therapy include pancreatic duct diameter, duct strictures, pain severity, risk of malignancy, and operative risk [9]. Surgical intervention is most often indicated for chronic debilitating pain and frequent attacks resulting in hospitalization once refractory to conservative and medical treatment [5,10]. Pancreaticoduodenectomy was once the standard operation for patients with chronic pancreatitis, however, other procedures such as duodenum-preserving pancreatic head resections and its variants have been introduced with good long-term results [1].

Operative management results in better long-term pain relief and mitigates the long-term risk of pancreatic cancer in patients with chronic pancreatitis. Lateral pancreaticojejunostomy or the modified Puestow procedure is the most common technique employed in the surgical management of chronic pancreatitis. This procedure provides definitive pain control and prevents progression of pancreatic damage when the cause is obstruction. However, hereditary pancreatitis has variable outcomes and surgery may not halt the progression of the disease [11]. It is best suited for chronic pancreatitis with calculi and pancreatic ductal dilatation > 8 mm [11,12]. Given our patient’s history of intractable, debilitating abdominal pain, frequent hospital admissions, pancreatic ductal dilatation of 10 mm, and numerous calculi along the length of this duct, we opted for a definitive surgical procedure. As such, we opted to employ the Rochelle-Partington modification of the Puestow procedure in the management of this patient.

The Rochelle-Partington modification is superior to the original Puestow procedure in terms of morbidity and mortality and has been more extensively studied than other drainage procedures [13]. Puestow and Gillesby developed a technique that combined a longitudinal opening of the pancreatic duct and an anastomosis to the small intestine with a pancreatic tail resection. Partington and Rochelle modified this procedure with an extended opening of the pancreatic duct and preservation of the pancreatic tail [1]. 83.1% of patients gain pain relief with a 27.8% recurrence rate after this procedure. There was a 1.2% mortality and 19.1% morbidity rate for 524 patients who underwent the Puestow procedure according to the National Surgical Quality Improvement Program (NSQIP) database in a study that looked at the 30-day outcomes [14]. A study from the Journal of Pain Research in 2019 compared procedures (modified Puestow, Frey, Pancreatoduodenectomy/pylorus-preserving pancreatoduodenectomy, Beger, distal pancreatectomy, and total pancreatoduodenectomy) with a total of 116 patients, which found that pain relief was over 80% in all operations with each having varying rates of pain recurrence and complications except total pancreatoduodenectomy which had no recurrence but had a mortality rate of 33.3% [1]. There has been a similar regional case of chronic pancreatitis in Jamaica with an 8-year-old patient who underwent the Puestow procedure which successfully halted her symptoms [15]. Due to the rarity of chronic pancreatitis in the pediatric population, it is difficult to study and compare these outcomes in children. The Dutch Pancreatitis Study Group is currently working on a randomized trial evaluating early surgery versus optimal current step-up practice for chronic pancreatitis (ESCAPE) to improve future treatment of chronic pancreatitis with a focus on pain relief, pancreatic function, and quality of life, compared to the current step-up practice [16]. Post-procedure, persistent pain can occur due to failure to drain the pancreatic duct at the head of the pancreas.

Measurement of drain amylase levels on the third postoperative day suggested the development of a grade B postoperative pancreatic fistula which was managed conservatively. Postoperative pancreatic fistulae are associated with intra-abdominal sepsis, hemorrhage, wound infection, bile leak, prolonged hospital stay, and death. Gland texture, histopathology, pancreatic duct diameter, and intraoperative blood loss can all be used to estimate the risk of developing a postoperative pancreatic fistula [13]. In a case of chronic pancreatitis, with a large duct diameter and minimal intraoperative blood loss, the risk of fistula formation is low.

After 20 days, our patient developed adhesional small bowel obstruction in the common limb which required surgical exploration. A loop of jejunum was used to perform two new anastomoses, to the Roux and Y limbs respectively, effectively bypassing the obstruction. Postoperative complications and the need for re-operation are relatively common in the surgical management of chronic pancreatitis in children. Complications reported include wound infection, hemorrhage, postoperative pancreatic fistula, bowel obstruction, splenic infarction, and recurrent pancreatitis [17]. Repeat surgery is most often indicated for recurrent pancreatitis [2].

However, the modified Puestow procedure has been widely reported as safe and reliable in the management of chronic pancreatitis in children [8,9]. Abdominal pain is significantly improved or even resolved in approximately 60% of patients irrespective of etiology [18]. Despite many challenges in the postoperative period, this patient has not experienced any recurrence of pancreatitis. After three years since surgery, the patient remains pain-free, well-nourished, and leads a normal life without any exocrine or endocrine insufficiency. We believe that the Rochelle-Partington modification of the Puestow procedure is safe and effective in the treatment of chronic pancreatitis in the pediatric population. It is important that surgeons retain a high index of suspicion for complications so that they can be identified and managed early.

## Conclusions

Whether the modified Puestow procedure is superior to other drainage procedures for chronic pancreatitis in the young is unknown. However, it is a safe and viable option in the management of chronic pancreatitis in children and adolescents where there is a significant disease burden. Currently, surgical treatment is associated with the preservation of exocrine and endocrine function, sustainable pain control, major improvement in quality of life, and reducing their long-term risk of developing cancer.
